# Uni-hemispheric hyperperfusion in the early postictal state: case report

**DOI:** 10.1186/s12883-020-01665-9

**Published:** 2020-03-24

**Authors:** A. Velasco Gonzalez, C. Schülke, B. Buerke

**Affiliations:** 1 Department of Clinical Radiology and Neuroradiology, University Hospital Muenster, Albert-Schweitzer-Campus 1, Building A1, 48149 Muenster, Germany; 2 Department of Clinical Radiology, University Hospital Muenster, Albert-Schweitzer-Campus 1, Gebäude A1, 48149 Muenster, Germany

**Keywords:** Stroke-mimics, Seizures, CT perfusion, Hyperperfusion, Case report

## Abstract

**Background:**

In the emergency setting of acute ischemic stroke, seizures have been reported in up to 4% of patients. In the absence of arterial occlusion, seizures may also cause abnormalities in CT perfusion in 78% of cases when the time window from onset to imaging is short. Both hyperperfusion and hypoperfusion in the postictal state have been described. Also, though rarely reported, postictal perfusion changes can be uni-hemispheric. In these cases, perfusion maps should be analyzed thoroughly, since perfusion reconstruction software relies heavily on a “normal” contralateral perfusion status.

**Case presentation:**

A 39-year-old man was found on the ground with a minor head injury. On admission, his reactions were generally slow, but there were no other neurological symptoms, and blood pressure was low. The patient had a history of primary generalized epilepsy and admitted to dropping off his anti-epileptic medication. He was transferred to the radiological department for imaging but shortly before began to experience generalized onset tonic-clonic seizures which were brought under control by intravenous therapy with 10 mg diazepam. After approximately 15 min, a multimodal CT scan was performed, revealing marked changes in the perfusion of the brain hemispheres and posterior fossa, with sharp delimitation at the midline. Blood gas analysis was congruent with respiratory acidosis. Clinically, the patient remained awake without developing any new symptoms. He gradually recovered over the following 3 h and, against our medical recommendation, discharged himself from the hospital.

**Conclusions:**

To the authors’ knowledge, this is the first report of an early postictal state describing sharply delimited uni-hemispheric hyperperfusion and hemispheric alteration of the cerebellum with an equally split rhombencephalon. Surprisingly, these changes were not associated with any focal neurological signs. To prevent misdiagnosis of perfusion alterations in seizures, radiologists and neurologists should be aware of the limitations of CT perfusion maps and software reconstructions. Novel use of CT perfusion reconstruction using peak enhancement helped in identifying the cerebral pathology.

## Background

In the emergency setting, CT perfusion is commonly evaluated for patients presenting with stroke symptoms. Whether associated with acute arterial intracranial occlusion or not, seizures have been reported in up to 4% of cases [1]. In the absence of arterial occlusion, seizures may also cause abnormalities in CT perfusion in 40% of cases, increasing to 78% when the time window from onset to imaging is short [2, 3]. Both hyperperfusion and hypoperfusion in the context of postictal states have been described. Nevertheless, hyperperfusion changes appear to occur more frequently when imaging ensues within 3 h after the seizures [2, 3]. In some cases, postictal perfusion changes are uni-hemispheric, mostly contralateral to the clinical deficits. These changes have been reported as hemispheric hypoperfusion [4, 5] or hyperperfusion [3, 6]. In such cases, the perfusion maps should be analyzed thoroughly, since perfusion reconstruction software relies heavily on a “normal” contralateral perfusion status. Thus, it can be a challenge to determine which of the areas of hyperperfusion or hypoperfusion indicated on perfusion maps are pathological.

We report the case of a young patient experiencing generalized onset tonic-clonic seizures shortly before performing a multimodal CT including perfusion imaging. With massive unilateral perfusion changes, this unique case not only describes the hemodynamic division of the brain after generalized seizures but also the limitations of perfusion maps, which tend to detect ischemia over hyperperfusion.

## Case presentation

A 39-year-old man with a history of opioid abuse and primary generalized epilepsy was found on the ground with a minor head injury. On admission, his reactions were generally slow, but there were no other neurological symptoms. The patient had refused to take his prescribed anti-epileptic medication [levetiracetam 750 mg twice daily]. Due to the patient’s condition and uncooperativeness, no further information as to the effect of the medication or the presence of compliance problems could be obtained. (R1.2) In view of the patient’s low blood pressure, 1 L of IV Sterofundin® ISO was administered. Blood tests for acute drug consumption were negative. A multimodal CT scan [non-contrast CT (NCCT), CT angiography, and CT perfusion (CTP)] were ordered. Shortly before the scan, the patient began to experience generalized onset tonic-clonic seizures which were brought under control by intravenous therapy with 10 mg diazepam. After approximately 15 min, a multimodal CT scan was performed. Arterial blood gas analysis revealed severe hypercapnia (pCO_2_: 72.8 mmHg), hypoxia (pO_2_: 22.4 mmHg), acidosis (pH: 7.24), a slightly elevated bicarbonate level (30 mmol/l), and normal lactate (1 mmol/L). Clinically, the patient remained awake without any focal neurological deficits. He gradually recovered over the following 3 h and, against our medical recommendation, discharged himself from the hospital.

**Fig. 1:**
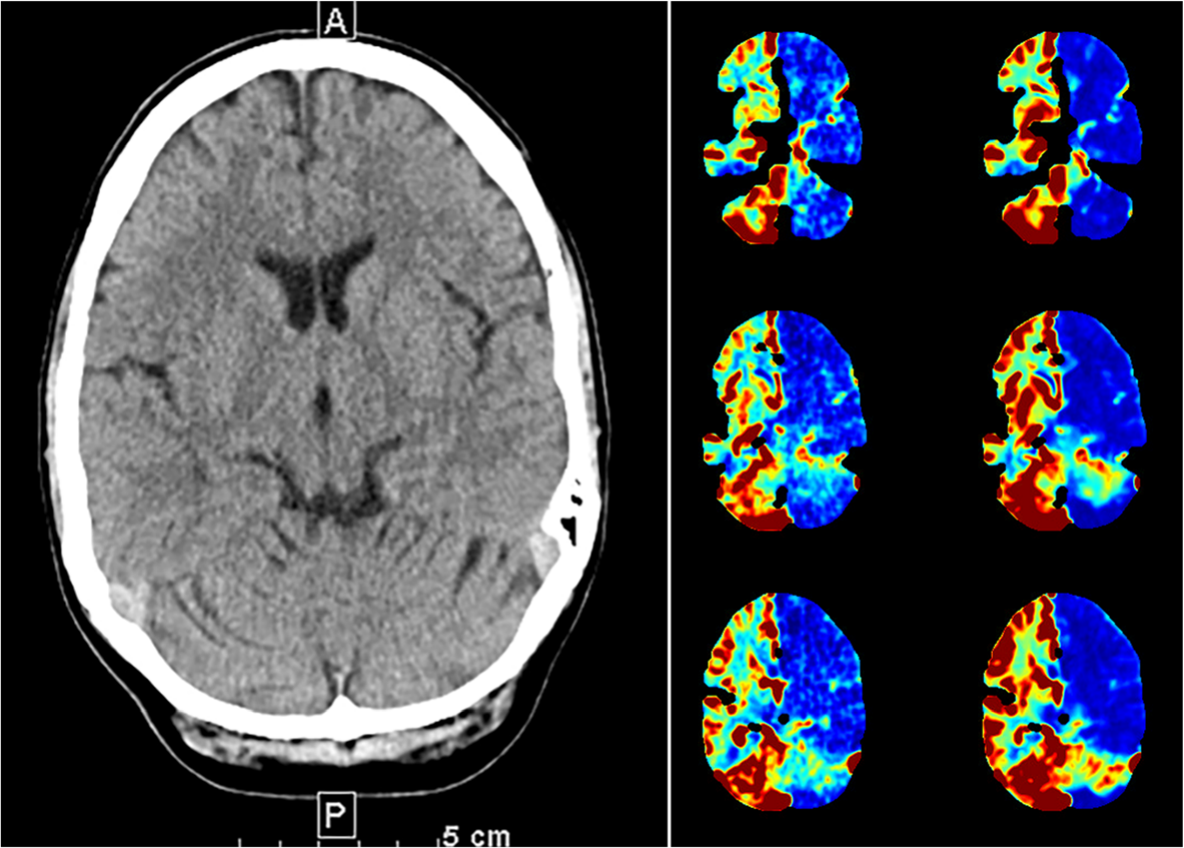
On the right, the non-contrast CT (NCCT) at the level of the basal ganglia. There were no signs of infarction or edema in the supratentorial and infratentorial structures. The corresponding relative cerebral blood flow (rCBF) and relative cerebral blood volume (rCBV) maps, at three different levels, are depicted on the left. The maps revealed a significant decrease in flow and volume in the left hemisphere, including the cerebellum, left midbrain, and pons, but flow and volume were preserved in the right hemisphere

**Fig. 2:**
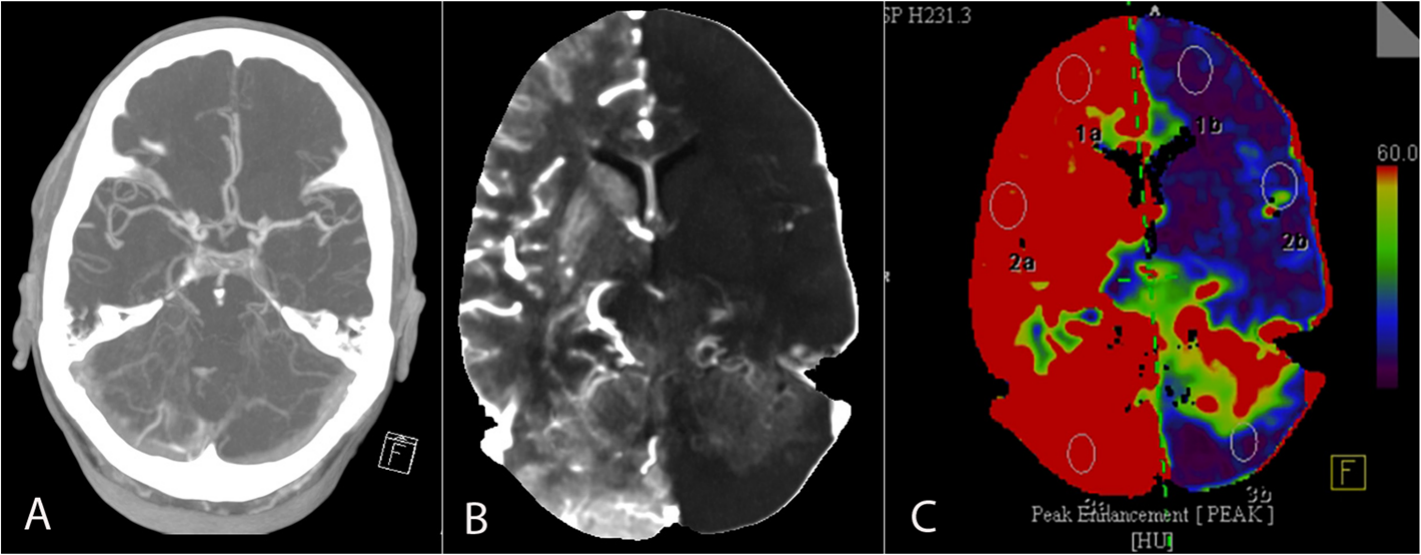
**a** Depicts the axial maximum intensity projection (MIP) reconstruction of the CT angiogram. Both middle cerebral arteries and the basilar artery were patent. There were also no signs of venous thrombosis. **b** shows the MIP reconstruction of CT perfusion from the source imaging. In **c**, the perfusion map obtained at the same level illustrates peak enhancement instead of cerebral blood flow or volume (Fig. 1). With no signs of arterial occlusion, the peak enhancement was nine times higher in the right hemisphere than in the left, including the left cerebellum, midbrain, and pons

There were marked changes in perfusion of the brain hemispheres and posterior fossa, with sharp delimitation at the midline. Imaging analysis revealed discrepancies between the features on the NCCT, relative cerebral blood flow (rCBF), and relative cerebral blood volume (rCBV) maps (Fig. 1). There was no infarct demarcation on the NCCT scan and no vessel occlusion on the CTA. Nevertheless, qualitative examination of the maps revealed a significant decrease in flow and volume in the left hemisphere but normal flow and volume in the right hemisphere. Further CT perfusion reconstruction using peak enhancement (Fig. 2a-c) revealed a peak nine times higher in the right hemisphere than in the left hemisphere as a result of vasodilatation and hyperperfusion [Hounsfield Units (HU) in temporal lobe: right (141), left (17.16); frontal lobe: right (99 HU), left (6.5 HU); cerebellum: right (201 HU); left (12 HU)] with no differences in the time to peak enhancement.

## Discussion and conclusions

Both hyperperfusion and hypoperfusion have been described in the context of the postictal state [2, 7]. Changes in brain perfusion may be a complex dynamic process from a little before from the onset of neuronal activity, through the early to the late postictal state, where normalization of seizure-related perfusion changes can be seen [7–9]. Nevertheless, hyperperfusion changes appear to occur more frequently when imaging ensues within 3 h after seizures [2, 3]. Hyperperfusion areas may include not only the epileptogenic focus but the adjacent or contralateral cortical regions, where ictal discharges are propagated [8]. It should be noted that these perfusion changes may also be seen in the cerebellum, usually contralateral to the supratentorial changes, in the form of cerebellar hypoperfusion (crossed cerebellar diaschisis) [5] or hyperperfusion [6]. In addition, hypercapnia (pCO2 > mmHg) and oxygen desaturation (< 90%) are not uncommon in patients with partial seizures and secondary, generalized convulsions and can lead to cerebral hyperperfusion [10, 11].

At first glance, the rCBF and rCBV maps (Fig. 1) could be misinterpreted as global hypoperfusion of the left hemisphere. In contrast, brain density on the NCCT was average. This would indicate that the left hemispheric hypoperfusion cannot be genuine. At this point, the limitations of CTP software reconstruction, which relies heavily on the assumption of “normal” contralateral perfusion, should be considered: CT-perfusion reconstructions for rCBV and rCBF maps tend to intensify the representation of ischemia over hyperperfusion. In addition, the CTA (Fig. 2a) depicted all intracranial arteries without any occlusion and further serves to rule out a differential diagnosis of atypical perfusion changes exceeding the arterial territories as in venous thrombosis.

We opted for another method of perfusion reconstruction in an attempt to gain a better picture of the hyperperfusion changes on the right side, which appeared to be underestimated on the rCBF and rCBV maps. The map of peak enhancement (Fig. 2c: maximum value in Hounsfield Units achieved) was a better means of demonstrating the global effect of capillary vasodilation in the right hemisphere but even so did not escape showing an “artificial”, i.e., not real hypoperfused left hemisphere. In summary, the perfusion maps relativized their values to those for the contralateral side of the brain. Thus, when one side is hyperperfused, the flow on the opposite side, by comparison, appears to be lower (hypoperfused). This does not represent genuine ischemia but rather is an artifact of the reconstructions, and the only true pathological finding of the CT-perfusion in this case was right hemispheric hyperperfusion.

Changes in arterial blood gases have also been described during convulsive attacks: pO_2_ decreases, pCO_2_ increases, and pH decreases. Such changes produce cerebral vasodilatation and have therefore been associated with high CBF [11, 12]. Our patient had severe hypercapnia (pCO_2_: 73 mmHg) that could be expected to lead to global cerebral hyperperfusion, not unilateral changes alone. Thus, the pathological hyperperfusion seemed to be a consequence of the epilepsy rather than of the systemic gas alterations. Nevertheless, though very unlikely, the absence of an EEG to demonstrate that the epileptic focus was located in the hyperperfused right hemisphere leaves another theory open. As an alternative though unlikely option, the hemispheric hyperperfusion could have been caused by the systemic hypercapnia, while the epileptogenic focus would be in left hemisphere with associated 1) ipsilateral discharges extended across the left hemisphere; 2) decreased vasoreactivity in the post-ictal state. Accordingly, the only part of the brain capable of reacting to the hypercapnia producing the hyperperfusion would be the right hemisphere with preserved vasoreactivity.

Finally, acute opioid consumption is associated with epileptic seizures in 8.5% of cases and may influence cerebral blood flow globally or focally [13, 14]. More recently, a reduction in rCBF after opioid administration has been described, affecting the different areas involved in the brain’s modulation of pain [15]. Despite a previous history of opioid consumption, tests for acute consumption were negative in the present case. Thus, opioid withdrawal as an epileptic trigger and as the cause of the brain perfusion changes can be dismissed.

In conclusion, then, severe perfusion changes can be detected on CT after seizures even without any intracranial arterial occlusion and may not be associated with focal neurological symptoms as in the present case. To our best knowledge, this is the first case published displaying these sharply delimitated hemodynamic changes down to the posterior fossa and serves as a clear example of the limitations of CT-perfusion reconstructions. The right hemispheric hyperperfusion was real but was only displayed by the peak enhancement reconstruction. Since detection of areas of hyperperfusion can be underestimated on perfusion map reconstructions of volume and flow, we would recommend including quantitative analysis of perfusion maps and adding peak enhancement reconstruction to the daily routine for hyperperfusion screening. Furthermore, with the increased use of multimodal CT for stroke taking place worldwide, stroke mimics should be more commonly seen in the early postictal state after epileptic seizures. Nevertheless, seizure patients tend to quickly be discharged from hospital, preventing us from being able to correlate the clinical findings, neurological findings, and EEG. Thus, we are at risk of misinterpreting the CT perfusion findings, independently of the potential limitations of the reconstructions, which means that a second reading after clinical and EEG findings are available is definitely required.

## Data Availability

The complete imaging study and medical records are available upon request with the approval of our ethical board.

## Abbreviations

CTP: CT perfusion
HU: Hounsfield units
MIP: maximum intensity projection
NCCT: Non-contrast CT
rCBF: Relative cerebral blood flow
rCBV: Relative cerebral blood volume

## References

[1] Kim SJ, Kim DW, Kim HY, Roh HG, Park JJ. Seizure in code stroke: stroke mimic and iniital manifestation of stroke. Am J Emerg Med. 2019;37(10):1871–5. 10.1016/j.ajem.2018.12.051 30598373

[2] Gelfand JM, Wintermark M, Josephson SA (2010). Cerebral perfusion-CT patterns following seizure. Eur J Neurol.

[3] Payabvash S, Oswood MC, Truwit CL, McKinney AM (2015). Acute CT perfusion changes in seizure patients presenting to the emergency department with stroke-like symptoms: correlation with clinical and electroencephalography findings. Clin Radiol.

[4] Zaidi SA, Haq MA, Bindman D, Mathur S (2013). Crossed cerebellar diaschisis: a radiological finding in status epilepticus not to miss. BMJ Case Rep.

[5] Vyas S, Bhatia V, Dass G, Sankhyan N (2019). Macroscopic and microscopic perfusion changes in hemispheric status epilepticus with crossed cerebellar diaschisis. J Pediatr Neurosci.

[6] Shin WC, Hong SB, Tae WS, Seo DW, Kim SE (2001). Ictal hyperperfusion of cerebellum and basal ganglia in temporal lobe epilepsy: SPECT subtraction with MRI coregistration. J Nucl Med.

[7] Austein F, Huhndorf M, Meyne J, Laufs H, Jansen O, Lindner T (2018). Advanced CT for diagnosis of seizure-related stroke mimics. Eur Radiol.

[8] Lee HW, Hong SB, Tae WS (2000). Opposite ictal perfusion patterns of subtracted SPECT. Hyperperfusion and hypoperfusion. Brain.

[9] Zhao M, Suh M, Ma H, Perry C, Geneslaw A, Schwartz TH (2007). Focal increases in perfusion and decreases in hemoglobin oxygenation precede seizure onset in spontaneous human epilepsy. Epilepsia..

[10] Seyal M, Bateman LM, Albertson TE, Lin TC, Li CS (2010). Respiratory changes with seizures in localization-related epilepsy: analysis of periictal hypercapnia and airflow patterns. Epilepsia..

[11] Pollock JM, Deibler AR, Whitlow CT, Tan H, Kraft RA, Burdette JH (2009). Hypercapnia-induced cerebral hyperperfusion: an underrecognized clinical entity. AJNR Am J Neuroradiol.

[12] Caspers H, Speckmann E-J (1972). Cerebral pO2, pCO2 and pH: changes during convulsive activity and their significance for spontaneous arrest of seizures. Epilepsia..

[13] Mattoo SK, Singh SM, Bhardwaj R, Kumar S, Basu D, Kulhara P (2009). Prevalence and correlates of epileptic seizure in substance-abusing subjects. Psychiatry Clin Neurosci.

[14] McPherson RW, Koehler RC, Kirsch JR, Traystman RJ (1997). Intraventricular dexmedetomidine decreases cerebral blood flow during normoxia and hypoxia in dogs. Anesth Analg.

[15] Adriaens A, Peremans K, Waelbers T, Vandermeulen E, Croubels S, Duchateau L (2014). The effect of morphine on regional cerebral blood flow measured by 99mTc-ECD SPECT in dogs. PLoS One.

